# Cross-Modal Imaging in Noninvasive Identification of Histologic Features of Skin

**DOI:** 10.1001/jamadermatol.2025.4318

**Published:** 2025-11-05

**Authors:** Sarah T. Arron, Afton Cobb, Lilia M. Correa-Selm, Katherine M. Given, Manu Jain, David Pilkington, Jennifer Y. Wang, Michael Z. Wang

**Affiliations:** 1 Palo Alto Foundation Medical Group, Palo Alto, California; 2 Advanced Dermatology, Jackson, Wyoming; 3 Morsani College of Medicine, University of South Florida, Tampa; 4 Moffitt Cancer Center, Tampa, Florida; 5 Given Dermatology Institute, Menlo Park, California; 6 Department of Dermatology, University of California, San Francisco; 7 Memorial Sloan Kettering Cancer Center, New York City, New York; 8 Golden State Dermatology, Merced, California; 9 Stanford University School of Medicine, Stanford, California; 10 Golden State Dermatology, Walnut Creek, California

## Abstract

**Importance:**

Histopathology with light microscopy is the reference standard for cellular evaluation of solid tissue, but whether noninvasive cross-modal imaging of skin provides clinicians with comparable histologic information that may assist in clinical decision-making is not well known.

**Objective:**

To evaluate safety and effectiveness of cross-modal imaging in obtaining in vivo images, to demonstrate that images align with corresponding skin histopathology, and to evaluate the ability of blinded readers to accurately identify tissue features on cross-modal images.

**Design, Setting, and Participants:**

This observational diagnostic study included 1 visit and follow-up call. Eligible participants were adults aged 18 to 99 years seen for routine care in the outpatient dermatology setting with an indication for lesional skin biopsy. The study was conducted from October 20, 2022, to August 11, 2023, at 2 outpatient dermatology clinics in the US. Cross-modal images and skin biopsy were collected from 1 lesion per participant. Participants were randomized to training (40%) and validation sets (60%). Comparative readers used the training set to evaluate cross-modal images against hematoxylin and eosin histopathology to validate tissue features and train blinded physician readers. Comparative readers developed a performance test with the validation set; blinded physician readers were tested on cross-modal feature identification without access to other participant data.

**Exposure:**

All participants underwent cross-modal imaging before biopsy.

**Main Outcomes and Measures:**

The primary end points were 100% comparative reader agreement and validation of cross-modal image tissue features against histopathology and 90% or greater accuracy of blinded physician readers identification of primary tissue features against comparative reader annotation. Secondary end points included accuracy of blinded physician reader identification of secondary features and greater than 90% interrater agreement between blinded physician readers. The safety end point was the number of adverse events observed.

**Results:**

A total of 65 participants (median age, 69 [range 20-93] years; 41.5% female and 58.5% male; 1.5% American Indian or Alaska Native; 13.8% Hispanic or Latino; 86.2% not Hispanic or Latino; 98.5% White) underwent cross-modal imaging. Comparative readers achieved 100% consensus and validation of cross-modal histologic features compared with histopathology. Blinded physician reader accuracy for primary histologic features was 96.4% (95% CI, 94.2%-98.7%) and for secondary histologic features was 98.5% (95% CI, 98.1%-98.9%). Interrater agreement among the blinded physician readers was high (Fleiss κ: region, 0.94 [95% CI, 0.87-1.0]; feature, 0.93 [95% CI, 0.88-0.97]). No adverse events were reported.

**Conclusions and Relevance:**

The results of this study suggest the safety of cross-modal imaging and that trained physicians may accurately identify histologic features on cross-modal images, consistent with its US Food and Drug Administration–cleared role in assisting clinical judgment.

## Key Points

**Question:**

Can cross-modal noninvasive imaging obtain in vivo images that align with skin histopathology, and can blinded readers accurately identify tissue features on these images?

**Findings:**

In this diagnostic study, 65 participants undergoing skin biopsy were imaged with a novel cross-modal system. Expert readers used traditional hematoxylin and eosin histopathology to validate skin features identified on cross-modal imaging and to train blinded physician readers; in performance testing, blinded readers scored 96.4% accuracy in feature identification.

**Meaning:**

The results of this trial provide evidence that physicians may use cross-modal imaging in the noninvasive identification of histologic features of skin.

## Teaser

This study evaluates the safety and effectiveness of the cross-modal system in obtaining in vivo images that show tissue features, align with corresponding pathology images from biopsies, and evaluate the ability of blinded readers to correctly identify tissue features on images obtained with the system.

## Introduction

Histopathology with light microscopy is the reference standard for cellular evaluation of solid tissue, but it typically involves invasive tissue collection and time-consuming slide preparation. Noninvasive high-resolution skin imaging technologies such as optical coherence tomography,^1^ line-field confocal optical coherence tomography,^2,3^ high-frequency ultrasound,^4,5^ and reflectance confocal microscopy (RCM)^6,7^ have emerged and been cleared for clinical use in dermatology by the US Food & Drug Administration (FDA). These technologies have been used successfully in clinical applications such as cancer,^8,9,10,11,12,13^ inflammatory disease,^14,15,16^ and skin health.^17,18,19^ However, these systems generate grayscale images, and RCM generates en face–oriented images, necessitating specialized training for accurate interpretation.^20,21^ Additionally, challenges persist with the ease of device operation, as most commercially available embodiments are bulky and have large imaging tips, hindering practical use in a clinical setting.

Cross-sectionally scanned multimodal microscopy (cross-modal imaging) is an emerging technology that noninvasively visualizes skin components with subcellular resolution in a compact handheld device. It combines RCM and multiphoton microscopy (MPM) displayed in cross section.^22^ RCM uses a near-infrared laser to produce high-resolution images of the epidermis and superficial dermis, highlighting structures with high refractive indices such as melanin and keratin, allowing for reliable recognition of skin pathology descriptors.^6,23,24^ MPM generates color-contrast of endogenous fluorophores such as nicotinamide adenine dinucleotide, flavin adenine dinucleotide, and collagen using 2-photon autofluorescence and second harmonic generation, providing high-resolution histology-like images that visualize cellular structures and differentiate collagen from surrounding tissue without exogenous dyes.^25,26,27^

The cross-modal system investigated in this study is a noninvasive imaging device (VIO; Enspectra Health) that contacts the skin surface with freehand maneuverability and real-time image generation to support point-of-care applications. The system optically scans cross-sectionally to a depth of 0.3 mm into the superficial dermis. The system delivers low-power laser light of a single wavelength (780 nm infrared) into the skin in brief pulses. The resulting signals are acquired from the skin and assigned to 1 of 4 channels: RCM, 2-photon autofluorescence long and 2-photon autofluorescence short, and second harmonic generation. The resulting images are digitally generated with a field of view comparable with that of a standard light microscope at 20× and 40× magnification.^22^ The study was designed to evaluate a wide range of common skin lesions indicated for biopsy, ensuring the observation of fundamental histological features across lesion types encountered in typical practice. The objectives of this pivotal study were to evaluate the safety and effectiveness of the cross-modal system in obtaining in vivo images that show tissue features, align with corresponding pathology images from biopsies, and evaluate the ability of blinded readers to correctly identify tissue features on images obtained with the system.

## Methods

### Participants

This study was a prospective multicenter single-arm diagnostic study to evaluate the safety and effectiveness of the cross-modal imaging system. This study followed the Standards for Reporting of Diagnostic Accuracy (STARD) reporting guideline. Safety was defined as the absence of device-related adverse events, and effectiveness was defined as the ability of readers reviewing images from the system to identify histologic features consistent with biopsy-confirmed diagnoses. Eligible participants were adults aged 18 to 99 years seen for routine care in the outpatient dermatology setting with an indication for lesional skin biopsy. The study was conducted from October 20, 2022, to August 11, 2023. Participants were excluded if the lesion to be biopsied was located on the palms, soles, nails, eyelids, or mucosa; was in an area of dense hair, tattoo, or clinically significant abraded or ulcerated skin, or skin too tortuous for the investigational device to access; or if the participant had a known allergy or skin sensitivity to silicone, adhesives, or glycerin.

Participants enrolled in the study had lesions photographed clinically and with dermoscopy, then marked with a donut-shaped targeting sticker for cross-modal imaging prior to skin biopsy. Medical assistants who had received less than a day of in-person training to use the cross-modal system performed cross-modal imaging of lesional and corresponding normal-appearing skin. Glycerin was used as an immersion fluid for cross-modal imaging of lesions. Biopsies were collected as part of standard of care outside of the study, and the resulting hematoxylin and eosin (H&E) histopathology was interpreted without access to information about cross-modal imaging results. Wong-Baker Rating Scale data^28^ on a scale of 0 to 10, where 10 indicates the worst pain imaginable, was collected for cross-modal imaging for each participant. Participants received a safety follow-up phone call 1 week after imaging.

A minimum of 20 lesional and 5 nonlesional cross-modal images were obtained per participant. The highest quality 4 to 8 lesional cross-modal images per participant underwent reader analysis. Images were assigned by participant to the training set or validation set using a validated algorithm that selected from randomized assignments while ensuring minimum enrollment of demographics in each set (eFigure 1 in Supplement 1).

The training set is defined as the set of images and associated clinical information used by the investigators to learn comparisons with histopathology and the overall clinical impression. The validation set is defined as the set of images and associated clinical information used to formally validate cross-modal imaging and for a blinded performance test.

This study was conducted in accordance with the protocol, Good Clinical Practice Regulations (21 CFR Parts 50, 54, 56, and 812), and the study sites’ research policies and procedures. The study protocol and informed consent form filled by participants were reviewed and approved by Salus Institutional Review Board.

### Readers

Comparative readers (S.T.A., K.M.G., M.J.) were members of the study team with experience in evaluating skin histopathology. Two comparative readers had expertise in noninvasive high-resolution imaging (S.T.A., 7 years; M.J., 13 years). Blinded readers (A.C., L.M.C., J.Y.W.) were required to be licensed and actively practicing physicians with residency and/or fellowship training in dermatopathology or equivalent, 2 or more years of dermatopathology experience within the last 5 years, reading slides at least once per week (including Mohs histopathology), and self-identify as being proficient in dermatopathology.

### Comparative Reader Training

Cross-modal images were randomized by participant, 40% to the training set and 60% reserved for the validation set. Comparative readers had access to lesional photography, dermoscopy, lesional and nonlesional cross-modal images, and digital and glass slide histopathology to develop consensus on tissue features. Primary features were defined a priori in the study protocol (epidermis, dermis, pigmented cell, collagen, and blood vessel). Secondary features were identified and defined during consensus review of the training set (stratum corneum, hair shaft or follicle, solar elastosis, hyperkeratosis, nodule or nest of cells, atypia, and epidermal disarray). To establish an early estimation of successful comparative reader learning before beginning validation, a fraction (23%) of the training images were reserved for an informal comparative reader self-test and then incorporated into the overall training set. Images for the performance test were locked until the comparative reader self-test confirmed annotation. Annotated training set images were developed into the training module for the blinded physician readers.

### Blinded Reader Training

Live interactive training of blinded physician readers was performed. The modules included (1) science of cross-modal imaging, (2) health care clinician training (hands on), (3) identifying primary features in normal skin on cross-modal images, 4) Identifying secondary features in normal skin on cross-modal images, (5) identifying features in lesional skin on cross-modal images, and (6) physician training for VIO Imaging for Skin Tissue Assessment (VISTA) study tools. Criteria for identification of primary and secondary features on cross-modal imaging were provided (eTable 1 in Supplement 1). Live training took approximately 5 hours. Blinded physician readers were provided access to training materials.

### Validation

To develop the performance test, 2 comparative readers (S.T.A., K.M.G.) independently reviewed and annotated cross-modal images from the reserved validation set without access to any other clinical images or information. Because annotations were arrows to points of example features or regions in images containing multiple examples and broad regions, comparative reader annotations could differ while still being correct. To ensure consistency, the third comparative reader (M.J.) evaluated these blinded independent annotations in comparison with histopathology. All comparative readers then reviewed the adjudicated set of annotations with access to all participant data and histopathology until 100% consensus was achieved. Consensus annotations were included in the performance test; annotations were excluded if definitive support was not found in histopathology slides, if comparative readers disagreed on interpretations, or if tissue features were obscured by annotation. Because of the strict standard for inclusion as a consensus annotation, many excluded annotations were likely correct; therefore consistency of comparative reader annotation was not measured.

### Performance Test

The performance test was built from validation set images with unlabeled annotations of each of the primary and secondary features. Duplicate annotations were consolidated by random selection. Annotations were then selected from each participant to create test questions (eFigure 2 in Supplement 1). Blinded physician readers were trained as described previously and completed the performance test with access to training materials.

### Statistical Analysis

Statistical performance assessed via percent agreement and Cohen κ with 95% CIs; no P values or α-level tests were used. Data analysis was performed using Excel version 2307 (Microsoft), R version 4.3.1 (R Foundation), and SAS version 9.4 (SAS Institute). Accuracy was defined as percent agreement of with the consensus annotation answer key reported as a mean of the 3 blinded physician readers. Interrater agreement among the BRs was assessed using Fleiss κ statistics and separately calculated for region (2 choices: epidermis vs dermis) and feature (4 choices: no feature, pigment, collagen, vs blood vessel). Accuracy and interrater agreement metrics are reported with 95% CIs. The point-estimate target for overall accuracy of primary features was 90%. A generalized linear mixed model adjustment to account for correlation among multiple images obtained for the same participant, and for correlation of responses within each BR, had little effect on overall accuracy; therefore, results of individual features are reported without generalized linear mixed model adjustment.

## Results

**Figure 1.**
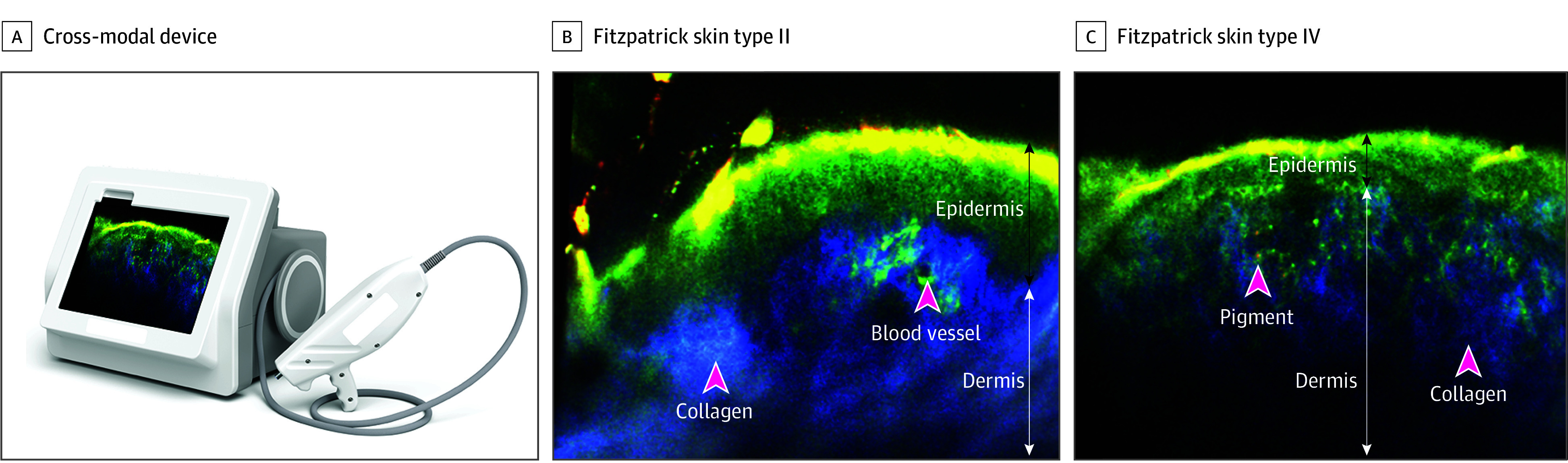
Cross-Modal In Vivo Imaging of Normal Skin (A) The cross-modal device. Cross-modal images of normal skin in participants with (B) Fitzpatrick skin type II and (C) Fitzpatrick skin type IV. Scale bar, 100 μm.

The study included 65 participants with a median age of 69 years (range 20-93 years), 41.5% female and 58.5% male; 1.5% American Indian or Alaska Native (1); 13.8% Hispanic or Latino (9); 86.2% not Hispanic or Latino (56); 98.5% White (64); 78.5% Fitzpatrick skin types I to III (51), and 27.7% lesions primarily diagnosed as basal cell carcinoma (18), 16.9% squamous cell carcinoma (11), 13.8% actinic keratosis (9), 12.3% seborrheic keratosis (8), and 12.3% nevi (8), 58.5% lesions located on the head and neck (38), 9.2% torso (6), and 32.3% limbs (21) (Table 1). The cross-modal device and imaging of normal skin are presented in Figure 1. Participants reported minimal discomfort during imaging (mean [SD] Wong-Baker Faces Pain Rating Scale, 0.15 [0.71]). No adverse events were reported during the study period.

**Table 1.** Participant Demographic Characteristics

Characteristic	No. (%)		
	Training set (n = 26)	Validation set (n = 39)	Total (N = 65)
Age, mean (SD) [range], y	66.9 (13.1) [41-92]	65.6 (18.6) [20-93]	66 (16) [20-93]
Sex			
Female	10 (38.5)	17 (43.6)	27 (41.5)
Male	16 (61.5)	22 (56.4)	38 (58.5)
Race^a^			
American Indian/Alaska Native	1 (3.8)	0	1 (1.5)
White	25 (96.2)	39 (100)	64 (98.5)
Ethnicity^a^			
Hispanic or Latino	2 (7.7)	7 (17.9)	9 (13.8)
Not Hispanic or Latino	24 (92.3)	32 (82.1)	56 (86.2)
Fitzpatrick skin type			
I	3 (11.5)	3 (7.7)	6 (9.2)
II	7 (26.9)	11 (28.2)	18 (27.7)
III	10 (38.5)	17 (43.6)	27 (41.5)
IV	5 (19.2)	6 (15.4)	11 (16.9)
V	1 (3.8)	2 (5.1)	3 (4.6)
VI	0)	0	0
Lesion anatomical location			
Head and neck	15 (57.7)	23 (59.0)	38 (58.5)
Torso	9 (34.6)	12 (30.8)	6 (9.2)
Limbs	2 (7.7)	4 (10.3)	21 (32.3)
Pathologic diagnosis			
Basal cell carcinoma	7 (26.9)	11 (28.2)	18 (27.7)
Squamous cell carcinoma	5 (19.2)	6 (15.4)	11 (16.9)
Actinic keratosis	3 (11.5)	6 (15.4)	9 (13.8)
Seborrheic keratosis	3 (11.5)	5 (12.8)	8 (12.3)
Nevus	3 (11.5)	5 (12.8)	8 (12.3)
Other^b^	5 (19.2)	6 (15.4)	11 (16.9)

^a^ Race and ethnicity were self-reported by participants through questionnaires. Race and ethnicity data were collected to enable accurate interpretation of results.

^b^ Other diagnoses include angioma, benign lichenoid keratosis, dermal scar, epidermal inclusion cyst, lentigo, melanoma, neurofibroma, sebaceous hyperplasia, subacute spongiotic dermatitis, and xanthogranuloma.

Images were randomized by participant to a training set (26 participants, 141 images) or a validation set (39 participants, 248 images). The demographics of participants and lesions randomized to the training and validation sets were similar. A total of 46% of the training set biopsies and 44% of the validation set biopsies were diagnosed on histopathology as basal or squamous cell carcinoma; a variety of other conditions were imaged including actinic keratosis, seborrheic keratosis, and nevi (Table 1).

Comparative readers used the training set to achieve consensus annotations on primary and secondary features with 100% concordance with the reference standard of glass slide histopathology. Comparative readers then validated consensus with the self-test (mean score: 100% primary; 97.3% secondary).

**Figure 2.**
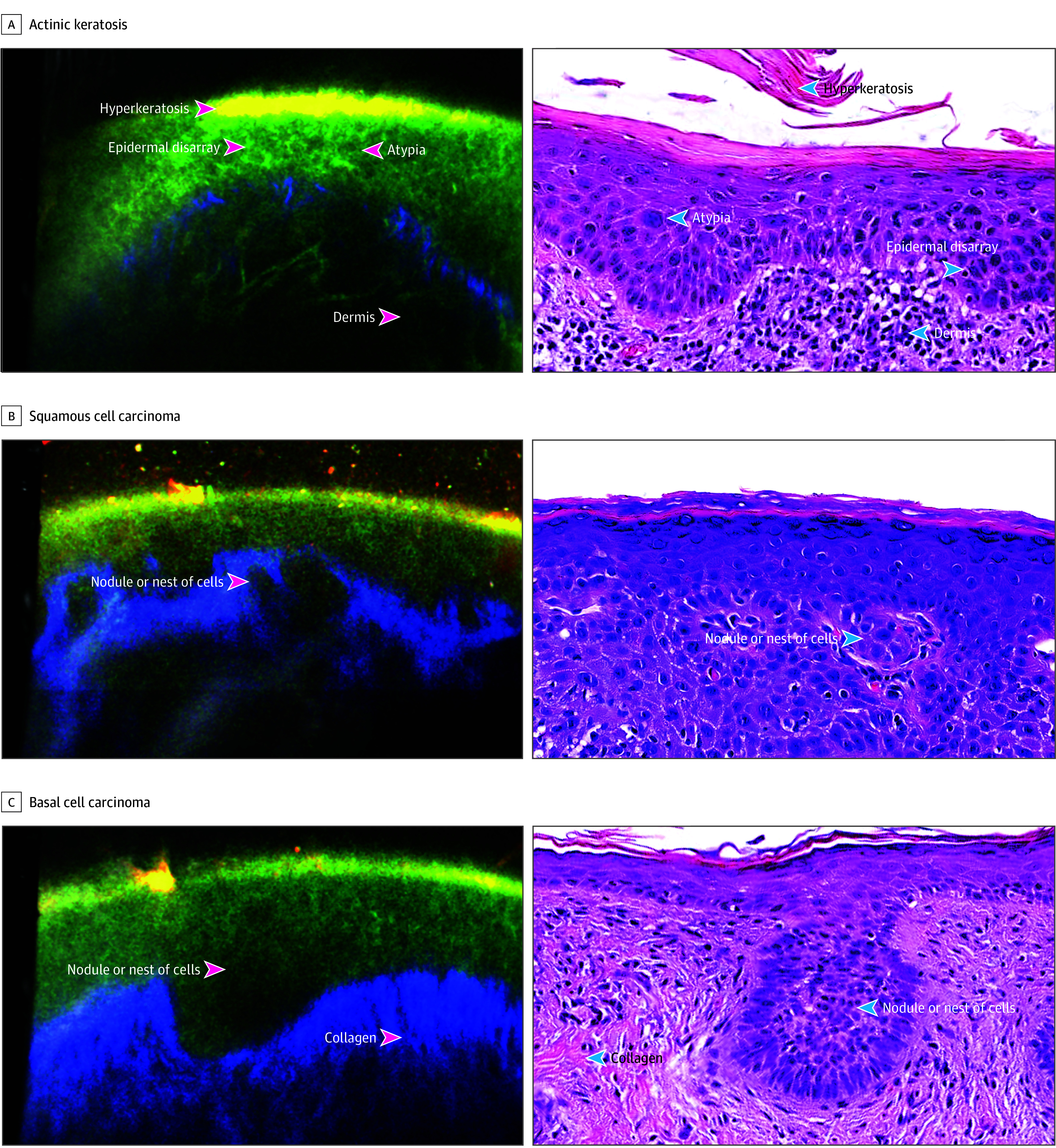
Cross-Modal In Vivo Imaging of Nonpigmented Lesions With Paired Histopathology Paired cross-modal and histopathology images of lesions shown for (A) actinic keratosis, (B) squamous cell carcinoma, and (C) basal cell carcinoma, superficial and nodular. Scale bar, 100 μm.

**Figure 3.**
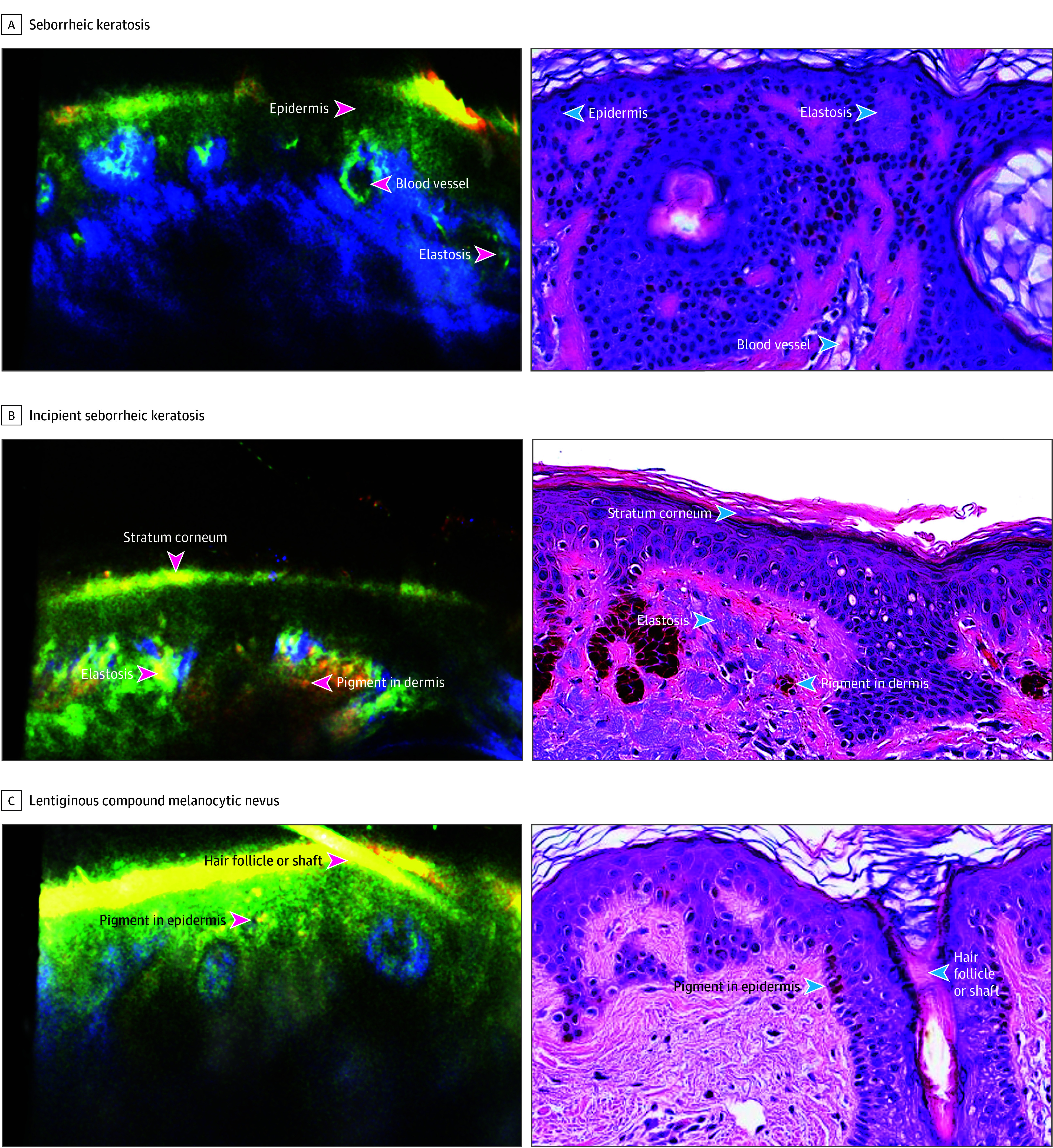
Cross-Modal In Vivo Imaging of Pigmented Lesions With Paired Histopathology Paired cross-modal and histopathology images of lesions shown for (A) seborrheic keratosis, (B) incipient seborrheic keratosis, and (C) lentiginous compound melanocytic nevus. Scale bar, 100 μm.

Cross-modal images in the validation set were then unlocked for comparative reader consensus annotation. The initial blinded comparative annotations achieved 91.3% (1275 of 1399) concordance with ground truth pathology; comparative readers were then unblinded and discussed images until 100% consensus was achieved. The performance test was developed from these annotated images and included 237 unique images from all 39 participants to create 450 test questions with 574 available points due to partial credit (Table 2). Histologic features were identified in the training and validation sets by comparative readers across a diversity of diagnoses in both nonpigmented (Figure 2) and pigmented lesions (Figure 3).

**Table 2.** Set Contents and Performance Test Results

Attribute	Images with attribute, No. (%)		Blinded reader accuracy, % (95% CI)
	Training set (n = 141 images)	Validation set (n = 248 images)	
Primary features and regions			
Epidermis only	137 (97.2)	241 (98.39)	96.0 (93.6-97.7)
Pigment in epidermis	46 (32.6)	38 (15.73)	93.5 (87.1-97.4)
Dermis only	25 (17.7)	13 (5.24)	100 (91.0-100)
Collagen in dermis	108 (76.6)	201 (81.05)	98.3 (96.7-99.3)
Pigmented in dermis	13 (9.2)	28 (11.29)	95.6 (89.0-98.8)
Blood vessel in dermis	14 (9.9)	13 (6.05)	87.2 (77.7-93.7)
Primary features total	NA	NA	96.2 (93.8-98.6)
Secondary features			
Stratum corneum	78 (55.3)	128 (55.65)	99.2 (97.1-99.9)
Hair shaft or follicle	27 (19.1)	15 (6.45)	97.8 (88.2-99.9)
Solar elastosis	35 (24.8)	69 (31.45)	99.1 (95.1-100)
Hyperkeratosis	19 (13.5)	15 (6.85)	100 (92.1-100)
Nodule or nest of cells	10 (7.1)	6 (2.82)	88.9 (65.3-98.6)
Atypia	24 (17.0)	26 (10.48)	95.6 (84.9-99.5)
Epidermal disarray	20 (14.2)	10 (4.03)	100 (88.4-100)
Secondary features total	NA	NA	98.5 (98.1-98.9)

Abbreviation: NA, not applicable.

Blinded physician readers were trained in the use and interpretation of the cross-modal imaging system with a 5-hour session before taking the performance test. Blinded physician readers achieved an adjusted mean score of 96.4% (95% CI, 94.2%-98.7%) accuracy on primary features, and each blinded physician reader exceeded the prespecified primary end point of 90% accuracy (Table 2). Unadjusted blinded physician reader performance test scores exceeded 90% across lesion and participant categories as well (eTable 2 in Supplement 1). Blinded physician readers achieved a mean score of 98.5% (95% CI, 98.1%-98.9%) accuracy on secondary features. Interrater agreement among blinded physician readers exceeded the 90% target (Fleiss κ: region, 0.94 [95% CI, 0.87-1.0]; feature, 0.93 [95% CI, 0.88-0.97]).

## Discussion

The results of this study suggest the safety and effectiveness of a novel cross-modal imaging system in obtaining in vivo images of lesional and normal skin. The device uses a combination of noninvasive imaging technologies to present cellular-scale images cross-sectionally and enables readers to accurately identify histologic features in the skin. There was little to no discomfort during imaging, and no adverse events were reported during the study follow-up.

Comparative readers with training and experience in evaluating both skin histopathology and RCM or MPM images achieved 100% consensus on primary features defined a priori in the study protocol and on secondary features identified in the training set. These consensus discussions were validated with a comparative reader self-test from reserved training images, demonstrating a mean accuracy of 100% on primary and 97.3% on secondary features; after follow-up discussion comparative readers came to 100% consensus on all features. All feature annotations were validated against the reference standard of histopathology. This high level of agreement underscores the robustness of our novel cross-modal imaging system, supporting its potential utility as a reliable tool to assist physicians in forming clinical judgments.

Interpreting cross-sectional composite images generated with cross-modal imaging is familiar for dermatologists and pathologists trained in vertically sectioned H&E histopathology. The list of histologic features chosen for this study (epidermis, dermis, pigmented cell, collagen, blood vessel, stratum corneum, hair shaft or follicle, solar elastosis, hyperkeratosis, nodule or nest of cells, atypia, and epidermal disarray) were included because their presence, absence, or form might assist in forming a clinical judgment. Additionally, only features that occurred in the training set with high enough frequency for sufficient comparative reader and blinded physician reader training were evaluated. Blinded physician readers with prior training and experience in dermatopathology were able to achieve 96% to 98% accuracy for primary and secondary features after 5 hours of training; each blinded physician reader exceeded the target primary end point of 90% accuracy. Blinded physician readers had near-perfect interrater agreement, exceeding the prespecified κ target of 0.9 or higher. These results demonstrate the effectiveness of our training protocol, and that physicians with experience in dermatopathology are able to reliably and quickly learn to use this system to identify the primary and secondary features listed in our study.

Familiarity, small footprint, and portability are other major benefits of the cross-modal imaging system over existing RCM and MPM devices. Medical assistants were trained in-person to operate the cross-modal system and capture images in the study setting, which highlights that users do not require advanced medical credentials to capture cross-modal images.

Cross-modal imaging is intended to be an adjunct device to assist in clinician decision-making. This study was not designed to assess or support claims of diagnostic accuracy to detect the diseases sampled in this study. Given this, we do not report binary classifier statistics such as the area under the receiver operating characteristic curves for individual features or diagnoses. The study supports an adjunct evidentiary standard: to assist in visualization of histology-like skin features that are validated by histopathology. The study was designed to image lesional skin prior to biopsy; therefore, we did not select specific diagnoses for inclusion. A strength of this approach is that we were able to demonstrate feature identification across a broad range of malignant and inflammatory skin conditions, but we were limited in our ability to characterize specific pathognomonic features. Future studies will expand on cross-modal findings in specific skin conditions and the ability of physicians to accurately identify these diagnosis specific features.

This study supports the potential for future integration of cross-modal imaging into routine dermatologic workflows. In future clinical settings, cross-modal imaging could complement traditional methods such as visual inspection and dermoscopy by providing histology-like information at the point of care. Its noninvasive and real-time capabilities may support prebiopsy assessment, guide treatment decisions, and potentially reduce unnecessary procedures. Additionally, future studies may explore its role in posttreatment monitoring for recurrence, in detection of early skin cancers that are visually indeterminate, and in cosmetic dermatology, where visualization of collagen integrity, elastosis, and pigmentary changes could inform personalized management strategies.

### Limitations

The limitations of the cross-modal imaging system include limited accessibility due to early commercialization, the imaging depth of 0.3 mm, which limits visualization to the epidermis and superficial dermis, and the small field of view, which necessitates freehand operation to explore larger regions. These technological limitations have demonstrated improvement over time. Cost is another potential limitation, although the cost of the device is not yet determined. Reimbursement in the United States for cross-modal has not been standardized, but cross-modal uses RCM, which has existing *Current Procedural Terminology* codes, payment, and coverage.

A major limitation of this study is that no study participants were Black or African American; 17.9% of the validation set participants identified as Hispanic or Latino. Black or African American participants were not excluded from the study, but none were enrolled as skin biopsies are less commonly performed in this population. Of note, in a previous small cross-modal imaging study, Black or African American participants were successfully imaged.^22^ Another important limitation is there were no study participants with Fitzpatrick skin type VI, although they were eligible. However, participants with Fitzpatrick skin types I to V were successfully imaged, with more than 20% of the validation set represented by Fitzpatrick skin type IV to V, indicating that skin pigmentation is not likely a barrier to the use of cross-modal imaging. Future research will expand our experience in imaging and interpreting images from a variety of skin conditions in diverse patient populations. Another limitation of the study is that it did not address clinician diagnostic accuracy. Finally, the study focused primarily on common benign and malignant skin lesions, which does not fully represent the spectrum of dermatologic conditions encountered in clinical practice. Future studies should aim to include a broader array of diagnoses to better assess the generalizability of cross-modal imaging.

## Conclusions

The results of this study suggest the safety of cross-modal imaging and that trained physicians may accurately identify histologic features on cross-modal images, consistent with its FDA-cleared role in assisting clinical judgment. The cross-modal imaging system has received FDA clearance (510(k) K232789) to acquire, store, retrieve, display and transfer in vivo images of tissue, including blood vessels, collagen, pigment, stratum corneum, hair shafts or follicles, solar elastosis, hyperkeratosis, atypia, and epidermal disarray, in and through epidermis for review by physicians to assist in forming a clinical judgment. This clearance was supported in part by the results of this study, which demonstrated high reader accuracy in identifying histologic features. Consistent with the design and execution of this study, clinical interpretation of cross-modal images must be performed by physicians who have dermatology or pathology medical qualifications with skin histology assessment training.

## Notes

Supplement 1. **eTable 1.** Criteria for Identification of Cross-Modal Image Features **eTable 2.** Performance Test Scores by Sub-Categories **eFigure 1.** Flow Diagram of Participant Involvement **eFigure 2.** Sample Performance Test Questions

Supplement 2.
Data Sharing Statement
