# Supplementary material for: Cross-Modal Imaging in Noninvasive Identification of Histologic Features of Skin
Source: JAMA Dermatol. 2025 Nov 5;162(2):115–23. doi: 10.1001/jamadermatol.2025.4318 (PMC12590393; doi:10.1001/jamadermatol.2025.4318)
Supplement: Supplement 1. — eTable 1. Criteria for Identification of Cross-Modal Image Features eTable 2. Performance Test Scores by Sub-Categories eFigure 1. Flow Diagram of Participant Involvement eFigure 2. Sample Performance Test Questions [file jamadermatol-e254318-s001.pdf]

Supplemental Online Content

Arron ST, Cobb A, Correa-Selm LM, et al. Cross-modal imaging in noninvasive identification of histologic features of skin. *JAMA Dermatol.* Published online November 5, 2025. doi:10.1001/jamadermatol.2025.4318

eTable 1. Criteria for Identification of Cross-Modal Image Features

eTable 2. Performance Test Scores by Sub-Categories

eFigure 1. Flow Diagram of Participant Involvement

eFigure 2. Sample Performance Test Questions

This supplemental material has been provided by the authors to give readers additional information about their work.

© 2025 Arron ST et al. *JAMA Dermatology*.

eTable 1. Criteria for Identification of Cross-Modal Image Features

**Skin Regions**	**Criteria for Identification of Region or Feature**
Epidermis	• Defined by keratinocytes; keratinocytes range from green to yellow to orange-red, depending on melanin content (2PL/2PS)<br>• Increased melanin in the epidermis shifts signal from green 2PS towards red 2PL
Dermis	• Subjacent dermis is primarily characterized by collagen in blue (SHG)
Dermato-epidermal junction (DEJ)	• Can be clearly defined or blurred depending on collagen signal intensity, angle of imaging, or presence of other features
**Primary Features**	**Criteria for Identification of Region or Feature**
Collagen	• In the composite color image, the SHG channel is represented by blue
Pigmented Cell	• Keratin in the epidermis shifts signal from green 2PS towards red 2PL<br>• Individual cells stand out on 2PL channel<br>• Increasing melanin signal pushes from green to yellow-green to red
Blood Vessel	• Blood vessels are dark, wrapped in blue collagen, sometimes having green speckles in vessel walls<br>• Absence of any signal can distinguish blood vessels from abnormal keratinocyte nodules or nests, which will have signal on the 2PL/2PS channels in the dermis
**Secondary Features**	**Criteria for Identification of Region or Feature**
Stratum corneum	• The outermost layer of the epidermis, composed of layers of flattened corneocytes<br>• Stratum corneum is a very bright yellow layer at the top of the epidermis (strong 2PL, even stronger 2PS)
Hair shaft or follicle	• Any component of the follicular unit, visualized at the bulb, isthmus, infundibulum, or shaft<br>• Hair shafts commonly seen, more than on H&E where they are often lost in processing<br>• Isthmus, infundibulum and shafts are typically the yellow-green of keratin<br>• The bulb and inner root sheath range from yellow to orange-red, both 2PL and 2PS<br>• Outer root sheath and shaft are green (like epidermis) due to increased signal in 2PS<br>• The bulb ranges from yellow to orange-red
Solar elastosis	• Irregular elastic fiber accumulation in the upper dermis, progressing to coarse clumps and tangles<br>• Solar Elastosis in the dermis is green-yellow from signal on the 2PL/2PS channel<br>• When solar elastosis is mixed with collagen (blue SHG) it ranges to aqua-white<br>• As elastosis increases, shifts to almost pure green-yellow with coarse clumps in the deeper dermis
Hyper-keratosis	• Thickened stratum corneum<br>• Often irregular contour<br>• Helpful to assess in context of epidermal thickness
Nodule or nest of cells	• A collection of epidermal cells displacing typical dermal structure<br>• Epidermal nodules or nests of cells have a subtle decrease in 2PS/2PL signal ('shadowed')<br>• May be delineated by wrapping collagen
Atypia	• Single focal enlarged keratinocyte.<br>• Dark enlarged nucleus highlighted by brighter rim of 2PL/2PS signal from keratin (and possibly NADPH)<br>• Compared to normal epidermis, epidermis with atypia is frequently more disorganized, thickened, and hyperkeratotic
Epidermal disarray	• Disorganized focus of keratinocytes with varied-sized cells, uneven keratin signal<br>• Atypical cells may be seen in the context of epidermal disarray

© 2025 Arron ST et al. *JAMA Dermatology*.

eTable 2. Performance Test Scores by Sub-Categories

		Primary Features			Secondary Features		
		Mean Score ± Standard Deviation<br>N = 3 Blinded Readers					
Condition	Basal Cell Carcinoma	97.7%	±	2.01%	98.6%	±	1.37%
	Squamous Cell Carcinoma	90.2%	±	4.49%	99.1%	±	0.79%
	Other	95.8%	±	1.77%	97.0%	±	0.00%
Location	Head and Neck	94.6%	±	1.77%	98.0%	±	0.00%
	Limb	98.1%	±	1.72%	100.0%	±	0.00%
	Torso	97.0%	±	2.71%	98.5%	±	1.31%
Fitzpatrick Skin Type	I	96.3%	±	4.24%	93.3%	±	2.89%
	II	98.4%	±	1.86%	100.0%	±	0.00%
	III	93.9%	±	3.03%	97.9%	±	0.92%
	IV	96.9%	±	2.61%	100.0%	±	0.00%
	V	97.6%	±	2.06%	100.0%	±	0.00%
Ethnicity	Hispanic or Latino	95.7%	±	3.27%	100.0%	±	0.00%
	Not Hispanic or Latino	96.3%	±	2.36%	98.3%	±	0.38%
Age	18-49	96.0%	±	3.15%	100.0%	±	0.00%
	50-64	97.9%	±	0.00%	100.0%	±	0.00%
	>65	96.0%	±	2.57%	98.1%	±	0.42%
Sex	Female	96.4%	±	2.41%	99.3%	±	0.63%
	Male	96.0%	±	2.49%	97.7%	±	1.15%

© 2025 Arron ST et al. *JAMA Dermatology*.

eFigure 1. Flow Diagram of Participant Involvement

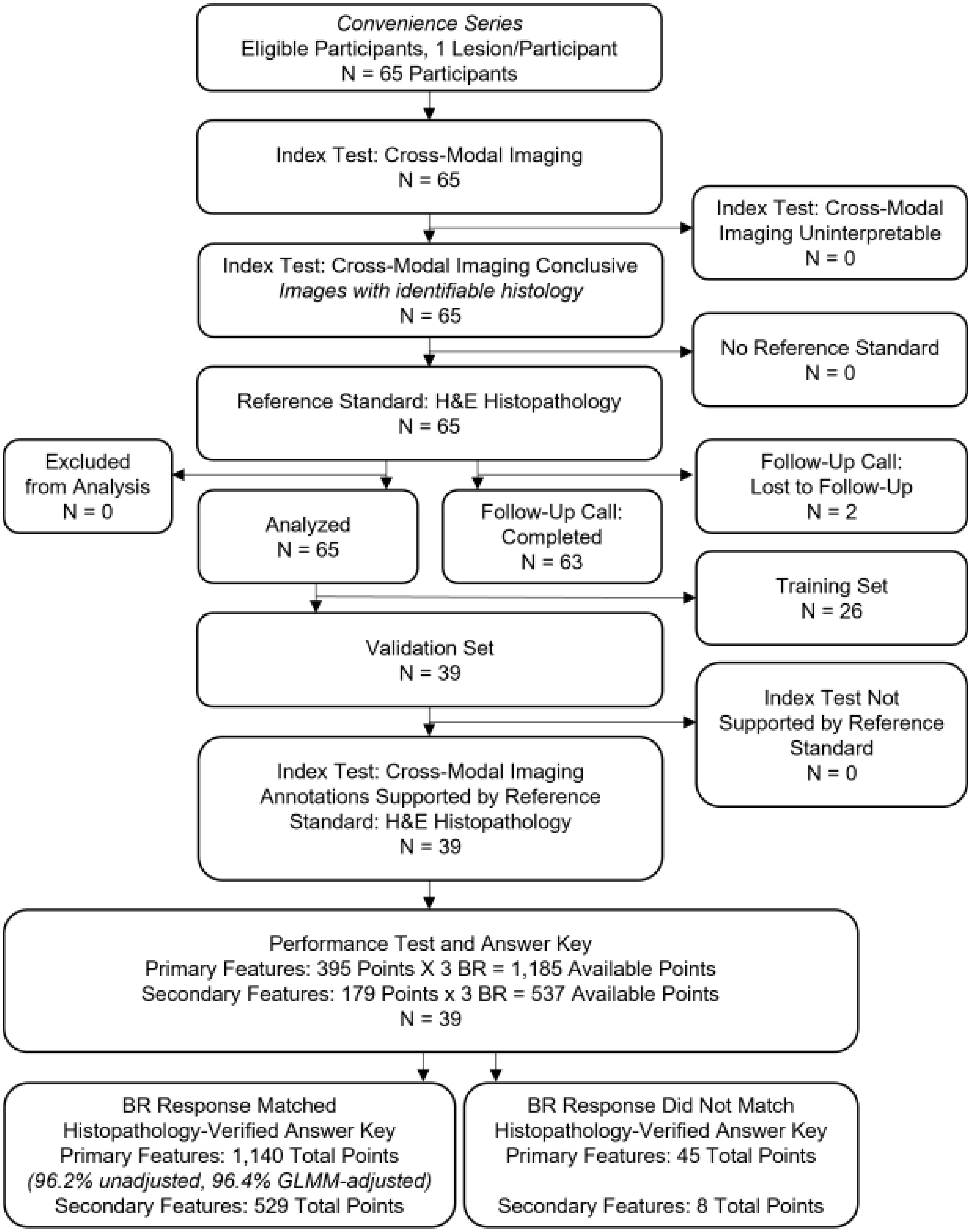

© 2025 Arron ST et al. *JAMA Dermatology*.

eFigure 2. Sample Performance Test Questions

Image	Question	Correct Answer
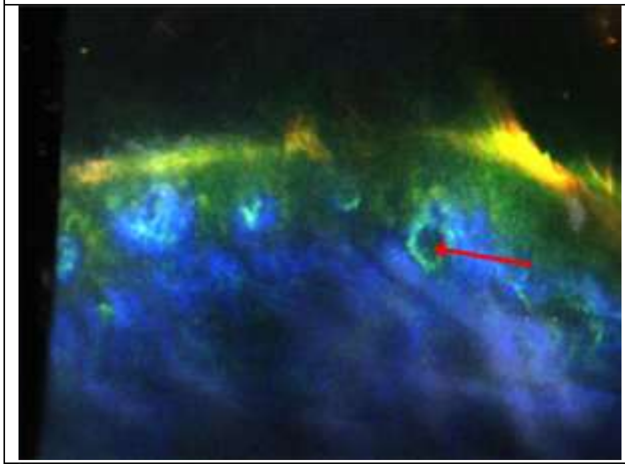	Primary Feature | Question 158<br>To what feature and region of skin is the tip of the arrow pointing? (feature/region)<br>A - No feature/Epidermis<br>B - Pigment/Epidermis<br>C - No feature/Dermis<br>D - Collagen/Dermis<br>E - Pigment/Dermis<br>F - Blood vessel/Dermis	F - Blood vessel/ Dermis
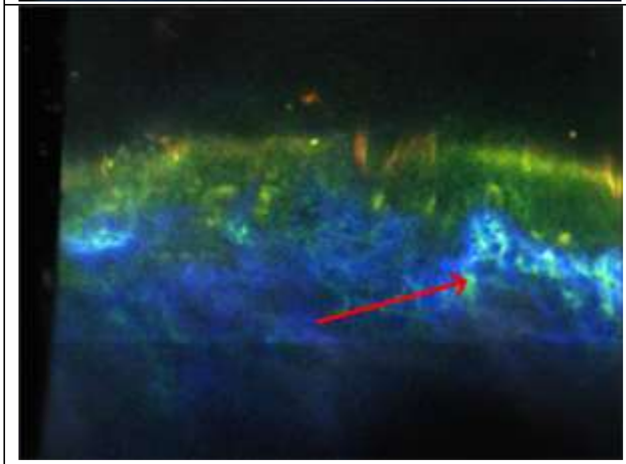	Secondary Feature | Question 392<br>To what feature is the tip of the arrow pointing?<br>A – Nodule or Nest of Cells<br>B – Solar Elastosis<br>C – Hair Shaft or Follicle<br>D – Stratum Corneum<br>E – Hyperkeratosis<br>F – Epidermal Disarray	B - Solar Elastosis
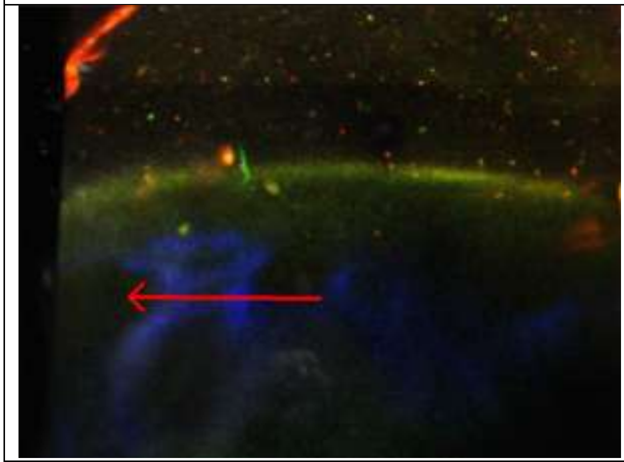	Secondary Feature | Question 366<br>To what feature is the tip of the arrow pointing?<br>A - Nodule or Nest of Cells<br>B - Solar Elastosis<br>C - Hair Shaft or Follicle<br>D - Stratum Corneum<br>E - Epidermal Disarray<br>F - Atypia	A - Nodule or Nest of Cells

© 2025 Arron ST et al. *JAMA Dermatology.*
