# Supplementary material for: Cross-Modal Imaging in Noninvasive Identification of Histologic Features of Skin
Source: JAMA Dermatol. 2025 Nov 5;162(2):115–23. doi: 10.1001/jamadermatol.2025.4318 (PMC12590393; doi:10.1001/jamadermatol.2025.4318)
Supplement: Supplement 2. — Data Sharing Statement [file jamadermatol-e254318-s002.pdf]

Data Sharing Statement

Arron. Cross-Modal Imaging in Noninvasive Identification of Histologic Features of Skin. *JAMA Dermatol*. Published November 05, 2025. doi:10.1001/jamadermatol.2025.4318

Data
Data available: Yes
Data types: Deidentified participant data
How to access data: info@enspectrahealth.com
When available: With publication

Supporting Documents
Document types: None

Additional Information
Who can access the data: These data may be requested by qualified researchers and will be provided after approval of the proposed use of the data.
Types of analyses: See previous statement.
Mechanisms of data availability: With an executed data access agreement.
Any additional restrictions: Proposals for data requests will be reviewed and considered for sharing after commercial release of this device and after publication of this manuscript. Data from this trial will be made available to others for at least 1 year.
